# Blueberry red ringspot virus genomes from Florida inferred through analysis of blueberry root transcriptomes

**DOI:** 10.1038/s41598-020-68654-3

**Published:** 2020-07-21

**Authors:** N. Saad, R. I. Alcalá-Briseño, J. E. Polston, J. W. Olmstead, A. Varsani, P. F. Harmon

**Affiliations:** 1 Department of Plant Pathology, University of Florida, Gainesville, FL USA; 2 Horticultural Sciences Department, University of Florida, Gainesville, FL USA; 3 The Biodesign Center of Fundamental and Applied Microbiomics, School of Life Sciences, Center for Evolution and Medicine, Arizona State University, 1001 S. McAllister Ave, Tempe, AZ 85287-5001 USA; 4 Structural Biology Research Unit, Department of Clinical Laboratory Sciences, University of Cape Town, Rondebosch, Cape Town, 7701 South Africa; 5 Present Address: Department of Plant Protection, Faculty of Agriculture, Universiti Putra Malaysia, Serdang, Selangor Malaysia

**Keywords:** Computational biology and bioinformatics, Plant sciences

## Abstract

A growing number of metagenomics-based approaches have been used for the discovery of viruses in insects, cultivated plants, and water in agricultural production systems. In this study, sixteen blueberry root transcriptomes from eight clonally propagated blueberry plants of cultivar ‘Emerald’ (interspecific hybrid of *Vaccinium corymbosum* and *V. darrowi*) generated as part of a separate study on varietal tolerance to soil salinity were analyzed for plant viral sequences*.* The objective was to determine if the asymptomatic plants harbored the latent blueberry red ringspot virus (BRRV) in their roots. The only currently known mechanism of transmission of BRRV is through vegetative propagation; however, the virus can remain latent for years with some plants of ‘Emerald’ never developing red ringspot symptoms. Bioinformatic analyses of ‘Emerald’ transcriptomes using de novo assembly and reference-based mapping approaches yielded eight complete viral genomes of BRRV (genus *Soymovirus*, family *Caulimoviridae*). Validation in vitro by PCR confirmed the presence of BRRV in 100% of the ‘Emerald’ root samples. Sequence and phylogenetic analyses showed 94% to 97% nucleotide identity between BRRV genomes from Florida and sequences from Czech Republic, Japan, Poland, Slovenia, and the United States. Taken together, this study documented the first detection of a complete BRRV genome from roots of asymptomatic blueberry plants and in Florida through in silico analysis of plant transcriptomes.

## Introduction

Blueberries are known to be infected with approximately 15 species of RNA and DNA viruses from 8 described and 2 unassigned genera^1^. Among the most common viruses affecting blueberry is blueberry red ringspot virus (BRRV) which causes red ringspot disease. Symptoms were originally described in New Jersey, United States and were observed on highbush blueberry in the 1950s but have since been reported in several other states, including Connecticut, Florida, Georgia, Michigan, New York and North Carolina^2,3^. Besides the United States, the presence of BRRV in cultivated blueberry also has been reported in eight countries including Belarus, Canada, Czech Republic, Japan, Poland, Slovenia and South Korea. Symptoms include faint red rings that are usually observed on new growth in early to late summer. Symptoms in early fall on older leaves also include red blotches that result from the coalescence of round red spots and rings. The red rings have centers with a pale green color and a diameter of 2–3 mm and 5–15 mm on leaves and stems, respectively^4^. The red spots and rings on leaves are typical disease diagnostic characteristics that are commonly observed on the upper leaf surface, but both sides of the leaves can be symptomatic depending on cultivar. BRRV is spread through vegetative plant propagation but can remain latent in asymptomatic plants for extended periods of time depending on the cultivar and age of the plants^3^. Arthropod pests have been investigated as potential vectors, but none currently are known to spread the virus.

BRRV belongs to the genus *Soymovirus* in the family *Caulimoviridae*^5,6^. BRRV has an 8.3 kb circular double-stranded DNA genome encapsidated in a nonenveloped, icosahedral particle with a diameter of 42–46 nm^7^ that can exist as a virion or form inclusion bodies in the nucleus or cytoplasm, respectively^8^. Members in the genus *Soymovirus* including BRRV have a genome that encodes for 8 proteins^9^.

Vast amounts of sequence data generated through various ‘omics’ approaches today open doors to many possibilities for post hoc analyses. One such possibility involves utilizing publicly available data sets from transcriptomics or genomics projects produced for other studies to data-mine for viral sequences. Plant transcriptome data generated by horticulturalists and other plant scientists have been used to search for viral sequences using in silico analyses. In one study, three nearly complete genomes (grapevine rupestris stem pitting-associated virus, grapevine pinot gris virus, and potato virus Y) were obtained from de novo assembled contigs of an existing grapevine transcriptome^10^. Later on, nearly complete genomes of bell pepper endornavirus and apple stem grooving virus were assembled in similar studies conducted using publicly available pepper, apple, and pear transcriptomes^11,12^. Although these studies have demonstrated the significant use of plant transcriptome data to gain insights into viral communities affecting plants, only one study has successfully obtained a complete viral genome from analyses of publicly available transcriptome data^13^. We used 16 root transcriptomes from eight clonal plants of the southern highbush blueberry (SHB) cultivar ‘Emerald’, an interspecific hybrid of *Vaccinium corymbosum* and *V. darrowi*, that were initially produced as part of a separate study conducted by the blueberry breeding program at the University of Florida to investigate blueberry response to soil salinity (Olmstead et al., unpublished).

In Florida, symptoms consistent with red ringspot disease have been observed but the complete genome sequence of BRRV has not yet been documented from within the state (only partial sequences JF917081–JF917085 are available in GenBank from an unpublished study). In this study, we identified and documented eight complete genomes of BRRV from Florida (Accession No: MN380630-MN380637) through bioinformatic analyses of root transcriptomes from asymptomatic blueberry plants of a cultivar known to develop red ringspot disease^14^. We additionally identify single nucleotide polymorphisms (SNPs) in each complete genome of BRRV assembled from the transcriptomes and determine phylogenetic relationships between the genomes of BRRV from Florida to those from other regions. This is the first report of red ringspot disease of blueberry in Florida and the first BRRV genome sequence from asymptomatic southern highbush blueberry.

## Materials and methods

### Source of transcriptome libraries

Softwood cuttings from a single mother plant of asymptomatic southern highbush blueberry cultivar ‘Emerald’ (*V. corymbosum* × *V. darrowi*), were rooted to produce eight clonal plants in a separate study (Olmstead et al., unpublished). The plants were used for the control treatment under optimal pH conditions for blueberry growth grown in a greenhouse during summer 2010. Total RNA was extracted from subsamples of root tissue from each 1-year old plant using a Plant/Fungi Total RNA Purification Kit (Norgen Biotek Corp., Thorold, ON) following the recommended manufacturer’s instructions. The RNA extracts were subjected to rRNA depletion using Epicentre Ribo-Zero™ rRNA Removal Kits (Epicentre, Madison, WI) followed by RNA library construction using Epicentre ScriptSeq v2 RNA-Seq (Epicentre, Madison, WI) library preparation kit according to the manufacturer’s protocol. Two sets of transcriptomes containing 100 nt paired end reads were generated in replicate sequencing reactions for each plant to produce a total of 16 libraries (eight libraries from each lane) using the Illumina HiSeq 2000 platform at the Interdisciplinary Center for Biotechnology Research (ICBR) Gene Expression Core, University of Florida.

### Transcriptome analysis

**Figure 1:**
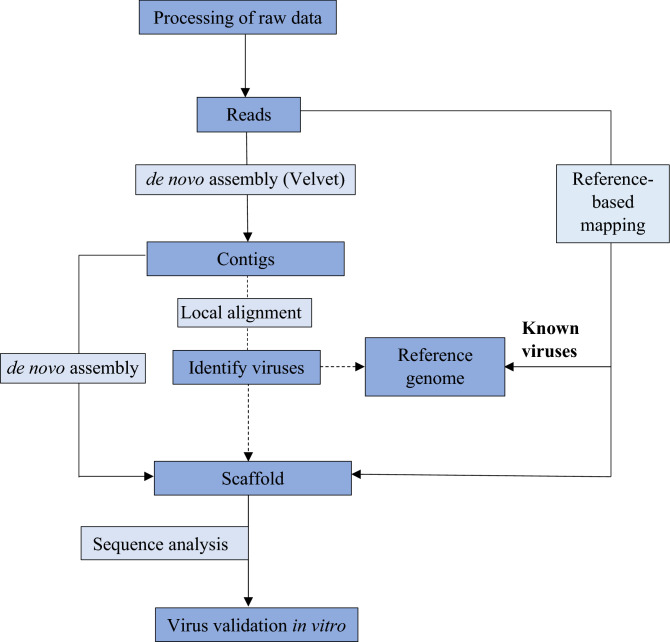
Transcriptome analysis pipeline used for data mining of viral sequences. Raw reads from each ‘Emerald’ blueberry library were processed by filtering the reads based on quality and trimming the adapter sequences. The processed reads were then assembled de novo to produce contigs. These contigs were aligned to a local plant virus database by BLASTx analysis to identify contigs with homology to viruses. Contigs with homology to blueberry red ringspot virus (BRRV) were then assembled to produce scaffolds to obtain the complete viral genome. Apart from de novo assembly, a reference based-mapping approach also was used to obtain the complete viral genome of BRRV from each transcriptome by mapping the reads to the BRRV scaffold. The presence of BRRV identified through this transcriptome analysis was finally validated in vitro by PCR. Adapted from^1^.

Paired end reads from each transcriptome (labelled as e9–e16) that corresponded to individual plants were analyzed according to the transcriptome analysis pipeline (Fig. 1). The reads were first de novo assembled using Velvet v1.2.09^15^ following quality filtering and trimming of adapters, resulting in 16 sets of contigs (Table 1). Only contigs with length ≥ 500 nt were compared to a local plant virus database^16^ by BLASTx^17^ with E-value of < 10^–5^. Contigs producing the same viral hits by BLASTx were assembled using Geneious assembler in Geneious v9.1.6 to produce scaffolds. These contigs and scaffolds were then compared to the sequences in the non-redundant GenBank protein database by using BLASTx. Complete viral genomes and average reads coverage were obtained by aligning the reads from each transcriptome to the de novo assembled scaffolds generated from the step above using Bowtie2 v2.3.0^18^. Analysis of SNPs of each assembled viral sequence were then performed using Geneious variant finder in Geneious v9.1.6 with default parameters (Minimum variant frequency: 0.25; maximum variant *p* value 10–6; minimum strand-bias *p *value 10–5 when exceeding 65% bias).

**Table 1:** No. of contigs from de novo assembly of reads following quality filtering and trimming of adapters obtained from blueberry root transcriptomes of southern highbush blueberry cultivar ‘Emerald’. Adapted from^1^.

Libraries/plant no.	No. of raw reads				No. of filtered reads				No. of contigs	
	Lane 1		Lane2		Lane 1		Lane2		Lane 1	Lane 2
	PE1	PE2	PE1	PE2	PE1	PE2	PE1	PE2		
e9	18,384,178	18,384,178	18,262,153	18,262,153	17,412,946	8,951,340	17,305,356	8,817,647	166,222	166,550
e10	16,945,940	16,945,940	16,748,977	16,748,977	16,106,215	8,315,739	15,942,835	8,140,610	76,832	76,222
e11	21,742,327	21,742,327	21,878,400	21,878,400	20,588,410	10,694,012	20,723,622	10,669,926	235,244	237,215
e12	17,737,529	17,737,529	17,606,724	17,606,724	16,946,223	8,696,389	16,849,588	8,555,510	229,856	229,269
e13	22,467,923	22,467,923	22,400,614	22,400,614	20,873,172	10,934,562	20,831,485	10,814,613	213,565	215,870
e14	12,151,144	12,151,144	12,051,449	12,051,449	11,406,139	5,928,887	11,326,171	5,833,078	127,012	126,363
e15	18,531,797	18,531,797	18,386,427	18,386,427	17,405,258	8,988,151	17,287,284	8,846,509	189,137	188,218
e16	17,948,384	17,948,384	17,946,456	17,946,456	16,870,384	8,738,272	16,890,100	8,673,219	168,999	170,908

*PE* paired end.

### Validation of BRRV

Total DNA was extracted from 30 mg of ground root ‘Emerald’ plant tissue using a modified CTAB procedure^19^. DNA extracted from a healthy plant of the ‘Southernbelle’ southern highbush blueberry cultivar was included as a negative control, while DNA was extracted from leaves of an ‘Emerald’ plant with red ringspot symptoms was included as the BRRV positive control in PCR. Each DNA sample was quantified using NanoDrop 2000 spectrophotometer (Thermo Fisher Scientific Inc. Waltham, MA). DNA samples [20 ng/µl] from individual ‘Emerald’ plants were used for the detection of BRRV. Fragment with an expected size of 549 bp derived from the transcriptional activator gene region was amplified using a set of primers (RRSV3F/RRSV4R)^20^ to validate the presence of BRRV in ‘Emerald’ plant tissue. PCR reactions were carried out by preparing a total of 20 µl reaction mixture containing 20 ng of total DNA, 3.1 mM MgCl_2_, 0.5 mM dNTP mix, 1.25 µM of forward and reverse primer, 1.25 µM Spermidine, and 0.625U *Taq* DNA Polymerase (New England Biolabs, Ipswich, MA). The cycling conditions for PCR were as follows: 94 °C for 3 min, 35 cycles of 94 °C for 30 s, 57 °C for 45 s, 72 °C for 45 secs, and final extension at 72 °C for 5 min. PCR products were resolved on a 1.0% agarose gel and the expected amplicon was purified using Illustra GFX PCR DNA and Gel Band Purification Kits (GE Healthcare Life Sciences, Chicago, IL). One amplicon from plant e11 was sequenced at Eurofins MWG Operon LLC (Eurofins Scientific, Luxembourg) to verify the identity of the amplicon.

### Sequence and phylogenetic analyses

Whole genome analysis was initially performed using the consensus sequence of BRRV (de novo assembled BRRV scaffold) obtained by scaffolding 75 contigs from all eight libraries, while the identity of the amino acid sequences of different ORFs with known protein functions used the in silico assembled BRRV genomes obtained from each library. Pairwise comparison between BRRV Florida sequences with other BRRV isolates from Czech Republic (HM159264), New Jersey (AF404509), Poland (JN205460), and Slovenia (JF421559) were computed by multiple alignment using MUSCLE^21^. Phylogenetic relationships between the amino acid sequences of these ORFs were inferred by the construction of phylogenetic trees in MEGA version 7.0^22^ by neighbor joining method, using bootstrap tests with 1000 replicates.

## Results

### Transcriptome analysis for identification of viruses

**Figure 2:**
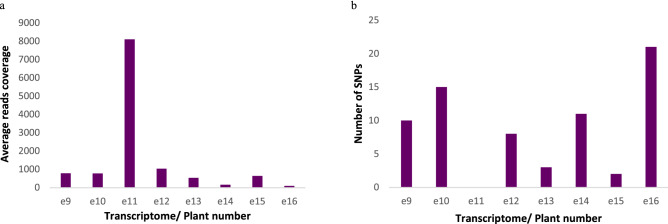
Alignment of reads from each transcriptome to the de novo assembled blueberry red ringspot virus scaffold using Bowtie2 implemented in Geneious 9.1.6. to obtain the (**a**) average read coverage and (**b**) no. of SNPs in each transcriptome using variant finder in Geneious v9.1.6 with the default parameters. Adapted from^1^.

Plant virus sequences were identified through bioinformatic analysis of the existing transcriptome from blueberry roots of cultivar ‘Emerald’ that were not known to harbor any viruses. A total of 119,888 contigs [length ≥ 500 nt] were obtained from de novo assembly of reads from 8 transcriptomes. Comparison of the contigs and scaffolds generated from assembly of the reads to the local plant virus protein database by BLASTx produced the highest number of virus hits to BRRV, and only one hit to three other viruses tentatively belonging to the family *Partitiviridae* and *Rhabdoviridae*) (Supplementary information Table 1). Based on the BLASTx results, the longest scaffold (nt) with highest viral hits to BRRV was selected for further analysis*. *De novo assembly of reads from the transcriptome yielded a complete genome of BRRV (genus *Soymovirus*, family *Caulimoviridae*) a plant virus with an open circular ∼ 8.3 kb dsDNA genome. Based on the de novo assembly result, the full-length genome of BRRV was initially obtained by scaffolding 75 contigs ranging from 500 to 1956 nt in size, producing a consensus sequence (BRRV scaffold) of 8293 nt length. Reference-based mapping was then performed independently in each library by using the in silico assembled BRRV scaffold as a reference sequence that resulted in eight complete genomes of BRRV, each from an individual ‘Emerald’ plant. Each BRRV genome contained 8 ORFs; ORF I (movement protein), A, B, C, IV (capsid protein), V (reverse transcriptase), VI (translational transactivator) and VII. A total of 900,057 reads (1.7%) from eight ‘Emerald’ libraries (367,406,012 reads) were mapped to the BRRV scaffold. The number of reads mapped to BRRV scaffold did not correlate with the total reads obtained in each library, with the lowest and highest proportion of mapped reads derived from library e16 (0.02%) and library e11 (1.12%), respectively (Table 2). The mapping of reads from each library to the BRRV scaffold also showed that library e11 displayed the highest average reads coverage with 8 to 88 times more than other libraries, which is in line with the percentage of mapped reads (Fig. 2a). Furthermore, identification of SNPs in reads mapped to BRRV scaffold indicated that there were 2 to 21 SNPs present in all libraries, except for library e11 which did not display any SNP (Fig. 2b). These SNPs resulted in a total of 29 amino acid substitutions in five de novo assembled BRRV genomes, with a range of 2 to 13 substitutions identified in each BRRV genome. However, there were no amino acid substitutions identified in the BRRV genomes assembled from library e11, e13 and e15. In addition, there were insertions in the de novo assembled BRRV genomes from e10 and e16 libraries, which resulted in frameshift mutations in ORF B and TAV, respectively.

**Table 2:** No. and percentage of reads aligned to scaffold of blueberry red ringspot virus. The percentage of mapped reads were obtained by using the following equation (no. of mapped reads/total no. of reads × 100%). Adapted from^1^.

Libraries/plant no	Total no of reads	No of mapped reads	% of mapped reads
e9	46,540,462	13,339	0.02866
e10	42,116,082	63,519	0.15089
e11	55,345,060	622,031	1.12391
e12	45,679,471	84,076	0.18406
e13	55,634,226	44,187	0.07942
e14	30,322,048	12,758	0.04207
e15	46,545,303	52,544	0.11289
e16	45,223,360	7603	0.01681
Total	367,406,012	900,057	1.73865

NP: scaffold of putative new viral species of Potyviridae; BRRV: *Blueberry red ringspot virus.*

### Validation of BRRV in blueberry cultivar *‘*Emerald*’*

**Figure 3:**
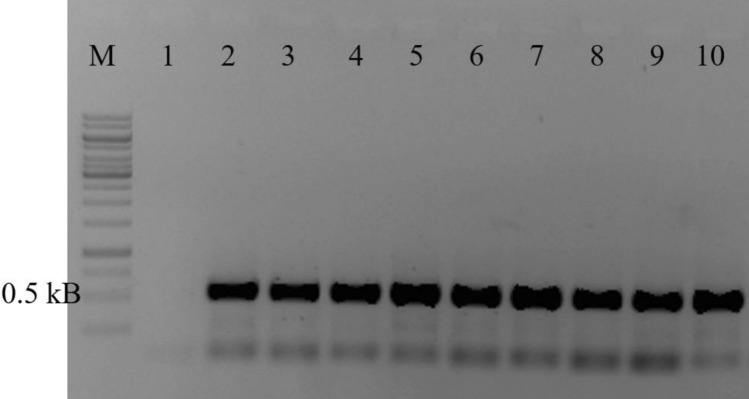
Validation of blueberry red ringspot virus in extracted DNA from ‘Emerald’ plant roots (*lane 2–9*) using virus specific primers (RRSV3F/R), producing an amplicon of 549 bp. Healthy ‘Southernbelle’ (*lane 1*) roots and red ringspot symptomatic ‘Emerald’ leaves (*lane 10*) were included as a negative control and positive control, respectively. M: 1kB DNA ladder Adapted from^1^.

The presence of BRRV in root samples of ‘Emerald’ was validated by PCR using the published virus specific primers^20^ in 100% of the root samples of ‘Emerald’ plants from which the transcriptomes were obtained, generating the expected 549 nt amplicon (Fig. 3). The amplicon sequence which was obtained from sample e11 produced highest identity (99%) to the transcriptional transactivator gene of BRRV isolate UF (JF917085) sampled in Florida from southern highbush blueberry in 2010 when compared to the GenBank nucleotide database by BLASTn. In addition, sequence alignment showed that the sequence had > 99% nucleotide identity to the BRRV consensus sequence.

### Sequence and phylogenetic analysis of BRRV

Although the genome organization of the BRRV-Florida (BRRV-FL) is similar with the previously deposited sequences, there are slight differences in the lengths of ORFs from BRRV-FL sequences (Table 3). ORF I of the BRRV-FL sequences contains the putative ‘transport domain’ (GNLKYGVIKFDV; aa 196–207), which is important for the movement of caulimoviruses within the host. ORFs A, B, and C of the BRRV encode for proteins with unknown functions, which are homologs of ORFs Ib, II, and III in soybean chlorotic mottle virus (SbCMV). The coat protein (CP) genes of the BRRV-FL isolates, encoded by the ORF IV, contain the RNA binding domain (CWICQEDGHYANEC; aa 411–425), which is a conserved motif among the caulimoviruses^8^. ORF V encodes for the putative reverse transcriptase gene containing the putative protease (YIDTGASLC; aa 31–39) and the core reverse transcriptase domains (YVDDIIIF; aa 356–363), which are conserved among caulimoviruses. Another conserved domain among the caulimoviruses, GLADTIY (aa 226–232), is also found in the ORF VI coding region of the BRRV-FL isolates, expressing the putative translational transactivator protein. The BRRV-FL isolates have the longest ORF VII, which is the least conserved regions among the caulimoviruses that encodes for an unknown protein function^8^ when compared to other published isolate sequences.

**Table 3:** Comparison of the nucleotide length of each open reading frame in the de novo assembled blueberry red ringspot virus genomes from Florida and other countries Adapted from^1^.

Isolates	ORFs								Total length
	I (MP)	A	B	C	IV (CP)	V (RT)	VI (TA)	VII	
CZ	1101	312	561	600	1488	2004	1284	462	8302
FL	1098	369	561	597	1488	2007	1284	477	8293
NJ	939	369	561	600	1461	1974	1287	429	8303
PL	939	369	561	594	1455	1974	1284	462	8265
SL	1110	369	561	588	1476	2043	1284	462	8299

*CZ* Czech Republic, *FL* Florida, *NJ* New Jersey, *PL* Polish, *SL* Slovenia, *CP* coat protein, *MP* movement protein, *RT* reverse transcriptase, *TA* transcriptional activator.

**Figure 4:**
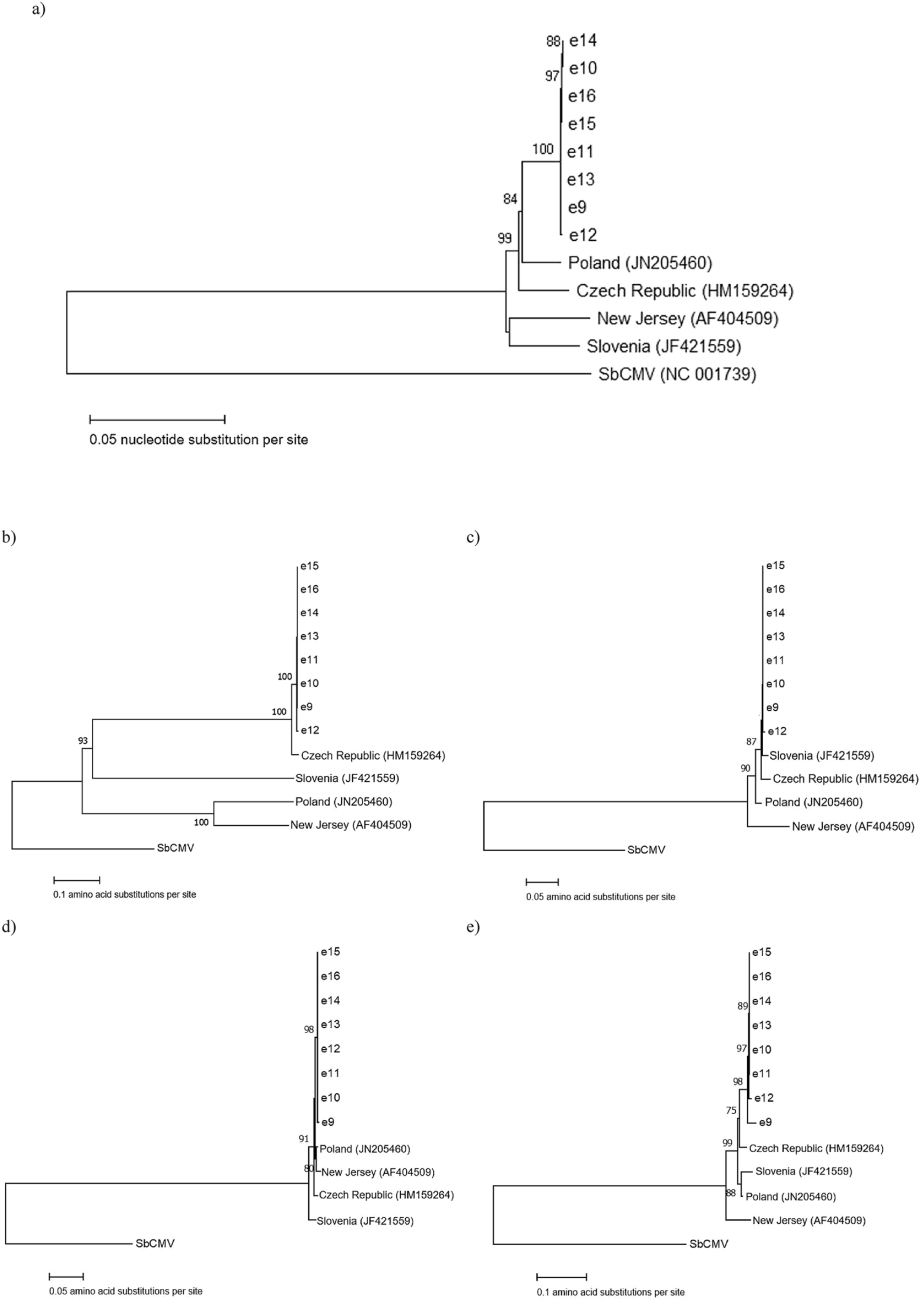
The evolutionary relationship inferred from aligned sequences of blueberry red ringspot virus from Florida (e9–e16) and other regions based on (**a**) whole genome, (**b**) CP, (**c**) MP, (**d**) RT and (**e**) TA, using SbCMV as an outgroup. The bootstrap consensus phylogenetic tree was constructed by Neighbor-joining method based on a matrix of pairwise distances estimated using a p-distance model with 1000 replicates, as shown by the value (%) at the branch nodes. *CP* coat protein, *MP* movement protein, *RT* reverse transcriptase, *TA* transcriptional activator. Adapted from^1^.

Whole genome analysis of the BRRV scaffold from this study and with other isolates showed that the BRRV sequence from Florida shared highest pairwise nucleotide identity (97%) to the published sequence of BRRV from Poland (JN205460) (Table 4). This is supported by multiple alignments of whole genome and different ORFs of BRRV sequences from Florida to those from other regions, which indicated that the BRRV-FL sequences had highest identity with the isolate from PL (97%) and lowest identity with isolates from SL and NJ (94–95%) (Table 5). Phylogenetic analysis of ORF V (RT) amino acid sequences showed that BRRV-FL sequences cluster with those of isolates from Czech Republic (HM159264), New Jersey (NC003138), Poland (JN205460), and distantly related to the isolate from Slovenia (JF421559) (Fig. 4). The ORF V (RT) of BRRV-FL isolates showed 99% aa identity to those from CZ, NJ, and PL and 97% aa identity to SL isolate. Further phylogenetic analyses between BRRV sequences using different ORFs (I, IV, V, VI and VII) with known protein functions showed that the local BRRV-FL sequences were clustered together in the same group (Fig. 4).

**Table 4:** Whole genome nucleotide alignment of blueberry red ringspot virus consensus sequence (de novo assembled BRRV scaffold) from Florida and other BRRV isolates by MUSCLE showing percent nucleotide identity between each isolate Adapted from^1^.

BRRV sequences	CZ	FL	NJ	PL	SL
CZ		96	94	96	95
FL	96		94	97	94
NJ	94	94		95	93
PL	96	97	95		96
SL	95	94	93	95	

*CZ* Czech Republic, *FL* Florida, *NJ* New Jersey, *PL* Polish, *SL* Slovenia.

**Table 5:** Whole genome nucleotide alignment of eight blueberry red ringspot virus sequences from Florida and other regions by MUSCLE showing percent nucleotide identity between each sequence.

BRRV sequences	NJ	SL	CZ	PL	e12	e10	e9	e14	e11	e13	e15	e16
NJ		93.27	93.73	94.55	93.78	93.73	93.78	93.78	93.82	93.82	93.82	93.78
SL	93.27		94.65	94.95	94.43	94.44	94.44	94.44	94.52	94.52	94.50	94.45
CZ	93.73	94.65		96.00	96.34	96.32	96.35	96.35	96.41	96.41	96.37	96.35
PL	94.55	94.95	96.00		96.55	96.52	96.56	96.56	96.63	96.63	96.59	96.58
e12	93.78	94.43	96.34	96.55		99.77	99.78	99.81	99.88	99.88	99.84	99.82
e10	93.73	94.44	96.32	96.52	99.77		99.78	99.92	99.87	99.87	99.90	99.89
e9	93.78	94.44	96.35	96.56	99.78	99.78		99.82	99.88	99.88	99.84	99.82
e14	93.78	94.44	96.35	96.56	99.81	99.92	99.82		99.91	99.91	99.95	99.93
e11	93.82	94.52	96.41	96.63	99.88	99.87	99.88	99.91		100.00	99.94	99.92
e13	93.82	94.52	96.41	96.63	99.88	99.87	99.88	99.91	100.00		99.94	99.92
e15	93.82	94.50	96.37	96.59	99.84	99.90	99.84	99.95	99.94	99.94		99.95
e16	93.78	94.45	96.35	96.58	99.82	99.89	99.82	99.93	99.92	99.92	99.95	

*CZ* Czech Republic, *FL* Florida, *NJ* New Jersey, *PL* Polish, *SL* Slovenia.

## Discussion

There are several approaches to obtain viral metagenomes, including the utilization of total RNA or DNA, virion-associated nucleic acids (VANA) extracted from virus-like particles (VLPs), double-stranded RNAs (dsRNA), virus-derived small interfering RNAs (siRNAs), and data mining using available NGS sequence data (e.g., transcriptome or genome database)^23,24^. It was previously demonstrated that plant mRNA libraries can be utilized for host plant studies as well as providing a source for viral metagenome studies^10–13,24^. Data mining of virus sequences was conducted in this study by using available blueberry root transcriptomes generated from one blueberry genotype that is being used in the blueberry breeding program in Florida, the ‘Emerald’ blueberry cultivar. In this study, we have exploited the availability of transcriptomes generated from blueberry roots for in vitro validation of latent virus infection.

Analysis of eight transcriptomes from eight clonally propagated ‘Emerald’ plants has led to the assembly of eight complete genomes of BRRV (8293 nt). These results provide the first complete genome of BRRV from Florida. Analysis of the assembled reads from each library using BRRV scaffolds from overlapping contigs have allowed us to determine the number of mapped reads and average of reads coverage. One library, e11, was shown to contain a significantly greater number of mapped reads, and average reads coverage, compared to other libraries, which suggests the presence of high virus transcripts in the corresponding plant. In addition, the absence of SNP in e11 library might also be attributed to the higher number of viral associated contigs and reads derived from this library in the BRRV consensus sequence obtained from de novo assembly of contigs pooled from all eight libraries, which was subsequently used as a reference sequence for mapping of reads from each library. The mutation rates in each BRRV genome assembled from each library based on the identified number of SNPs were between 0 and 0.25%, implying low genetic diversity among these sequences. The SNPs associated with amino acid substitutions identified in five BRRV genomes may or may not cause any changes in the protein functions. However, frameshift mutations identified in ORF B and TAV of two de novo assembled BRRV genomes could possibly affect the protein functions encoded by these ORFs. While the function of ORF B is yet to be discovered, TAV is known to play an important role as a transactivator/viroplasmin and was recently shown to be responsible for intracellular movement of caulimovirus virions of the cauliflower mosaic virus^25^. Viruses are known to exist as quasipecies in nature, with variations amongst viral sequences normally identified using Sanger sequencing. However, this conventional approach would have required a large amount of additional work, especially given the size of BRRV genome assembled in this study (8293 nt). Thus, the SNPs in each library were identified using Geneious variant finder which included parameters to appropriately identify real SNPs while filtering out variants resulted from sequencing errors. Additional sequence and phylogenetic analyses performed in this study to compare the identity and relationship between the genomes of BRRV from Florida to those from other regions showed that the BRRV sequences from Florida shared > 99% identity among each other. The high identity and low genetic diversity between these sequences are expected because the transcriptomes were obtained from plants that were clonally propagated. BRRV sequences from Florida were shown to be closely related to BRRV isolate sequences from Poland with 97% nt identity, and 94% nt identity with the one from New Jersey, implying that there could have been exchange of plant stock or germplasm between these regions.

Additional research is needed to determine if BRRV can integrate into the host genome. Members of all genera in the family *Caulimoviridae*, except the genus *Soymovirus,* are found as endogenous pararetrovirus sequence (EPRS)^26^. Additional efforts outside the scope of this project will be required to determine whether the BRRV sequences are integrated into the host genome or present in an episomal form. For other members of the *Caulimoviridae* family, this has been accomplished using rolling circle amplification and back to back primers^27,28^.

### Informed consent

Informed consent does not apply to this study.

## Supplementary information

Supplementary information
