# Supplementary material for: Blueberry red ringspot virus genomes from Florida inferred through analysis of blueberry root transcriptomes
Source: Sci Rep. 2020 Jul 21;10:12043. doi: 10.1038/s41598-020-68654-3 (PMC7374169; doi:10.1038/s41598-020-68654-3)
Supplement: Supplementary file 1 — Supplementary information [file 41598_2020_68654_MOESM1_ESM.pdf]

Supplementary information:

Blueberry red ringspot virus genomes from Florida inferred through analysis of blueberry root transcriptomes

Saad, N. [1], Alcalá-Briseño, R.I.[1], Polston, J.E.[1], Olmstead, J.W. [2], Varsani, A. [3,4], Harmon. P.F. [1]

1 Department of Plant Pathology, University of Florida, Gainesville, Florida, United States.

2 Horticultural Sciences Department, University of Florida, Gainesville, Florida, United States.

3 The Biodesign Center of Fundamental and Applied Microbiomics, School of Life Sciences, Center for Evolution and Medicine, Arizona State University, 1001 S. McAllister Ave, Tempe, AZ 85287-5001, USA

4 Structural Biology Research Unit, Department of Clinical Laboratory Sciences, University of Cape Town,

Rondebosch, Cape Town 7701, South Africa

Unedited gel pic

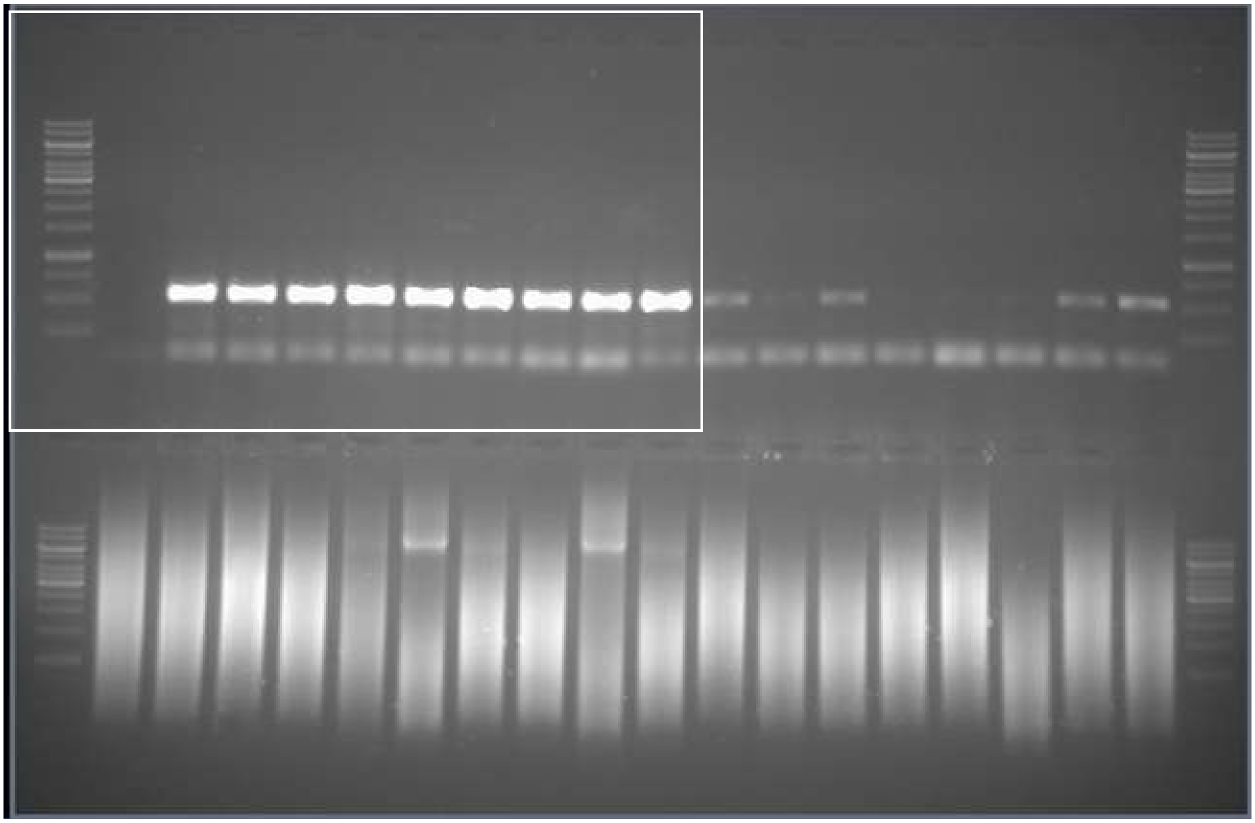

Supplementary Table 1. Virus hits obtained by BLASTx analysis of the contigs and scaffolds obtained from *V. arboreum* and *Vc x Vd* 'Emerald' to the non-redundant protein sequence database in GenBank. "adapted from dissertation by N. Saad, 2017, https://ufdc.ufl.edu/UFE0051785/00001"

Plant species	Contig/ Scaffold length (nt)	BLASTx	Family/ Genus	Region of the genome	Query cov (%)	Identity (%)	E-value
Vc x Vd 'Emerald'	8293	*Blueberry red ringspot virus**	*Caulimoviridae*	Complete genome	100	97	0
	992	Black grass varicosa-like virus	*Rhabdoviridae*	Polyprotein	70	37	6E-39
	861	Vicia faba partitivirus	*Partitiviridae*	Putative RdRp	89	40	3E-60
	755	Persimmon latent virus	*Rhabdoviridae*	RdRp	96	24	4E-08
	533	Persimmon latent virus	*Rhabdoviridae*	Pro-ala	26	47	3E-05

*Refers to scaffold. RdRp: RNA dependent RNA polymerase; Pro-ala: proline-alanine-rich protein.
